# A pilot study on using acupuncture and transcutaneous electrical nerve stimulation (TENS) to treat knee osteoarthritis (OA)

**DOI:** 10.1186/1749-8546-3-2

**Published:** 2008-02-29

**Authors:** Kazunori Itoh, Satoko Hirota, Yasukazu Katsumi, Hideki Ochi, Hiroshi Kitakoji

**Affiliations:** 1 Department of Clinical Acupuncture and Moxibustion, Meiji University of Oriental Medicine, Kyoto 629-0392, Japan; 2 Department of Orthopaedic Surgery, Meiji University of Oriental Medicine, Kyoto 629-0392, Japan

## Abstract

**Background:**

The present study tests whether a combined treatment of acupuncture and transcutaneous electrical nerve stimulation (TENS) is more effective than acupuncture or TENS alone for treating knee osteoarthritis (OA).

**Methods:**

Thirty-two patients with knee OA were randomly allocated to four groups. The acupuncture group (ACP) received only acupuncture treatment at selected acupoints for knee pain; the TENS group (TENS) received only TENS treatment at pain areas; the acupuncture and TENS group (A&T) received both acupuncture and TENS treatments; the control group (CT) received topical poultice (only when necessary). Each group received specific weekly treatment five times during the study. Outcome measures were pain intensity in a visual analogue scale (VAS) and knee function in terms of the Western Ontario and McMaster Universities Osteoarthritis Index (WOMAC).

**Results:**

The ACP, TENS and A&T groups reported lower VAS and WOMAC scores than the control group. Significant reduction in pain intensity (P = 0.039) and significant improvement in knee function (P = 0.008) were shown in the A&T group.

**Conclusion:**

Combined acupuncture and TENS treatment was effective in pain relief and knee function improvement for the sampled patients suffering from knee OA.

## Background

Osteoarthritis (OA) is the most common form of joint disease and most often affects the knee [1,2]. OA of the knee causes patients severe discomfort and a reduced ability to work [1,2]. Anti-inflammatory drugs used to treat the symptoms of this disorder usually have various side-effects [3]. When drugs are not adequately effective, replacement surgery is often recommended [4]. Patients with chronic pain increasingly seek alternative methods for pain relief, particularly transcutaneous electrical nerve stimulation (TENS) [5] and acupuncture [6-8]. TENS has the advantage of being efficacious, inexpensive, simple and essentially free of side effects. TENS may even be used at home by patients themselves due to its portability and simplicity. Several studies examined the efficacy of acupuncture and TENS treatment for such conditions; however, the results were inconclusive [5-8]. Acupuncture and TENS were found effective in treating pain and dysfunction in patients with knee OA [5-8]. A systematic review covering seven randomised controlled trials concluded that acupuncture and TENS were effective in reducing pain; however, differences in efficacy between acupuncture and TENS were inconclusive [5-8].

The present study aims to test whether TENS or a combined treatment of acupuncture and TENS is more effective than acupuncture or TENS alone for treating knee OA in older patients.

## Methods

### Patients

Outpatients aged 60 years or older with knee OA were recruited from the Meiji University of Oriental Medicine Hospital. The patients had been clinically and radiologically diagnosed of knee OA according to the criteria of the American College of Rheumatology. Further inclusion criteria for the present study were: (1) knee pain lasting for six months or longer; (2) no radiation of knee pain; (3) radiographic evidence of at least one osteophyte at the tibiofemoral joint (Kellgren-Lawrence grade ≥ 2); (4) normal neurological functions of lumbosacral nerve, including deep tendon reflexes, voluntary muscle action and sensory function; and (5) not receiving acupuncture treatment for more than six months. Exclusion criteria were: (1) major trauma or systemic disease; and (2) receiving conflicting or ongoing co-interventions. Patients under drug treatment were included if there had been no change in medicine and its dosage for one month or longer. This study was approved by the Ethics Committee of the Meiji University of Oriental Medicine.

All enrolled patients gave their written informed consent. According to a block randomised allocation table (generated by Sample Size, version 2.0, Int), the enrolled patients were allocated to (1) the control (CT) group, (2) the acupuncture (ACP) group, (3) the transcutaneous electrical nerve stimulation (TENS) group or (4) the acupuncture and TENS (A&T) group.

### Design

The design of this study was a randomly controlled clinical trial using a block randomised procedure. Each patient received a total of five treatments, once per week, and follow-up was measured for ten weeks after the first treatment.

### Treatment

#### Control (CT) group

The CT group patients did not receive any specific treatment, but when necessary, were allowed to use topical poultice containing methylsalicylic acid.

#### Acupuncture (ACP) group

The ACP group patients received acupuncture treatment at selected acupoints for 15 minutes on the OA affected knee. The selected acupoints are widely accepted for treating knee pain [6-13], namely *Liangqiu *(ST34), *Dubi *(ST35), *Zusanli *(ST36), *Yinlingquan *(SP9), *Xuehai *(SP10) and *Yanglingquan *(GB34). Disposable stainless steel needles (0.2 mm × 40 mm, Seirin Co Ltd) were inserted into the muscle to a depth of 10 mm using the 'sparrow pecking' acupuncture technique (alternate pushing and pulling of the needle) by acupuncturists who had four years of acupuncture training and three to eight years of clinical experience. When the subject felt dull pain or the acupuncture sensation (*de qi*) was achieved, the needle manipulation was stopped and the needle was left in place for ten more minutes.

#### Transcutaneous electrical nerve stimulation (TENS) group

The TENS group patients received treatment at the OA affected knee for 15 minutes from a single-channel portable TENS unit (model HV-F3000, OMRON Healthcare Co Ltd, Japan), which sends between two electrodes a premixed amplitude-modulated frequency of 122 Hz (beat frequency) generated by two medium frequency sinusoidal waves of 4.0 and 4.122 kHz (feed frequency). Surface disposable electrodes of 809 mm^2 ^and 5688 mm^2 ^were respectively placed on the site with the most tenderness and the opposite side of the site. Two electrodes were different in size (ratio: 1:7). The smaller one was placed on the site of tenderness. The intensity of TENS stimulation was adjusted so that a tingling sensation 2–3 times of the subject's sensory threshold was produced.

#### Acupuncture and transcutaneous electrical nerve stimulation (A&T) group

The treatment for the A&T group combined the treatments for the ACP and TENS groups. The patients received 15 minutes of TENS, and then 15 minutes of acupuncture treatment at the OA affected knee.

We confirmed that the patients in all groups did not receive any other co-interventions including analgesics, anti-inflammatory agents or topical hyaluronic acid injection during the study period.

### Evaluation

Primary outcome measures were: (1) pain intensity, quantified with a 10 cm visual analogue scale (VAS, 0 – 100 mm) and (2) pain disability measured with the Western Ontario and McMasters Universities Osteoarthritis Index (WOMAC, 0 – 100 points) [14]. The WOMAC consists of 24 questions each with five possible responses.

The VAS scores were measured immediately before the first treatment and subsequently at one, two, three, four, five and ten weeks after the first treatment. The WOMAC scores were measured immediately before the first treatment and subsequently five and ten weeks after the first treatment. Each VAS and WOMAC score was measured immediately before treatment of the specified week.

### Statistical analysis

Repeated measures analysis of variance (ANOVA) was used to study the changes in the VAS and WOMAC scores in the three groups. Changes in the time course among groups were considered significant when the interaction was significant at a level of 0.013 (0.05/4). After detection of significant changes in the overall time course with repeated measures ANOVA, pair comparisons were detected with Bonferroni correction. StatView for Windows (version 5.0) or SYSTAT 10 (SYSTAT Inc) was used for the statistical analysis. The results with P values of less than 0.05 were considered statistically significant.

## Results

### Patients

A total of 32 patients (21 women, 11 men; aged 62 – 83 years) were randomly allocated to four groups for specific treatment. Before the treatments, no significant difference was found in baseline variables including age, disease, pain duration, and pain intensity VAS scores among the four groups.

**Figure 1:**
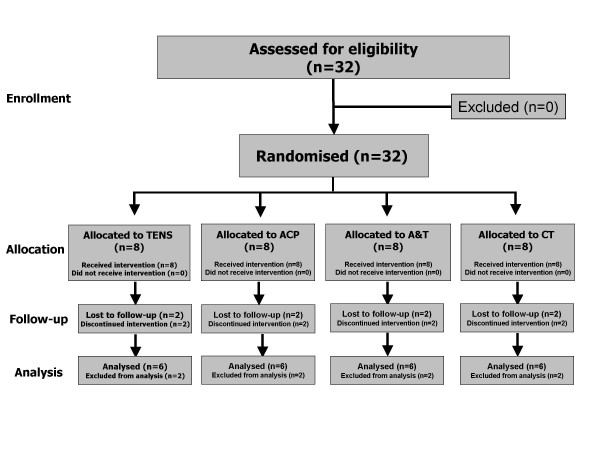
Patient participation flow in the study.

One patient in the ACP group, two patients in the TENS group, one patient in the A&T group, and two patients in the CT group dropped out as they had not responded to the respective treatment. In addition, one patient in the ACP group and one patient in the A&T group dropped out due to adverse effects (*i.e. *deterioration of symptoms). The total dropout rate was the same in every group. The analyses were performed on the 24 patients who completed the study and provided required information (Figure 1).

### Pain intensity VAS scores

The mean pain intensity VAS scores decreased in all groups during treatment, although the exact time courses varied (Table 1). In the A&T group, the pre-treatment (0-week) VAS score and 5-week VAS score were significantly different (P = 0.039). However, differences between the pre-treatment (0-week) scores and 5-week scores of the ACP, TENS and CT groups were not statistically significant.

**Table 1:** Pain intensity VAS scores

**Week**	**CT (n = 6)**	**ACP (n = 6)**	**TENS (n = 6)**	**A&T (n = 6)**
**0**	59.8 (18.5)	61.2 (12.3)	64.3 (10.3)	56.6 (12.6)
**1**	64.8 (14.4)	61.2 (11.8)	63.8 (7.0)	50.7 (10.4)
**2**	54.3 (8.3)	56.8 (13.6)	53.3 (10.3)	40.7 (8.7)
**3**	57.3 (10.8)	51.5 (14.6)	49.5 (10.6)	39.3 (19.1)
**4**	55.2 (10.8)	51.5 (15.8)	42.3 (15.2)	37.3 (19.1)
**5**	54.5 (8.7)	41.7 (10.6)	38.8 (13.3)	33.3 (11.7)*
**10**	49.3 (20.2)	43.0 (21.2)	53.5 (9.7)	41.3 (20.2)

Data are expressed as mean (SD).

* P = 0.039

During the first five weeks of treatment, while the ACP, TENS and A&T groups all reported lower mean pain intensity VAS scores than the CT group, only the A&T group gave a statistically significant reduction in VAS scores.

### WOMAC scores

The mean WOMAC scores decreased in all groups during treatment, although the exact time courses varied (Table 2). In the A&T group, the pre-treatment (0-week) WOMAC score and 5-week WOMAC score were significantly different (P = 0.008). By the end of the fifth week of treatment, the ACP, TENS and A&T groups reported lower WOMAC scores than the CT group. However, the differences between the treatment groups were not statistically significant.

**Table 2:** Western Ontario and McMaster Universities Osteoarthritis Index (WOMAC) scores

**Week**	**CT (n = 6)**	**ACP (n = 6)**	**TENS (n = 6)**	**A&T (n = 6)**
**0**	51.2 (8.5)	54.5 (9.5)	54.2 (15.1)	46.5 (12.9)
**5**	48.3 (8.2)	40.5 (8.2)	39.2 (7.4)	37.7 (16.3)*
**10**	50.8 (10.6)	45.2 (10.1)	46.2 (8.8)	40.2 (18.0)

Data are expressed as mean (SD).

* P = 0.008

## Discussion

Acupuncture and TENS are non-pharmacological treatment methods for a variety of pain conditions. This pilot study assessed the effects of acupuncture and TENS on knee OA for later clinical trials.

The ACP, TENS and A&T groups showed decreases in pain intensity (VAS scores) compared to the CT group during treatment (Table 1). The treatment groups showed lower mean WOMAC scores than that of the CT group at week 5 of treatment. These results suggest that acupuncture and TENS treatments have positive effects on the quality of life (QOL) of the knee OA patients, and that A&T treatment was significantly more effective in terms of WOMAC scores than other treatments (Table 2).

The present study demonstrated that acupuncture and TENS treatments were effective in pain relief. TENS is a common modality for treating musculoskeletal pain [15]. TENS excited large-diameter afferent fibres [16]. According to the gate control theory [17], TENS may stimulate the large-diameter afferent fibres, which may reduce the transmission of pain signals through the small nociceptive afferent fibres, thereby inhibiting pain discrimination and perception. TENS has been shown to produce antinociceptive effects similar to those of acupuncture [18,19] with slow onset and gradual offset that persists after the stimulation stops [20]. Acupuncture excited small-diameter afferent fibres [21,22]. Similar to descending inhibition and/or diffuse noxious inhibitory controls (DNICs) in the brain system, acupuncture may stimulate the small-diameter afferent fibres, which may reduce the transmission of pain signals, thereby inhibiting pain discrimination and perception [23]. Moreover, acupuncture is effective for improving knee function such as range of motion (ROM) and walking/climbing time [6-13]. It is possible that acupuncture affects tension and blood flow in the muscle. The present study demonstrated that acupuncture was effective in improving the QOL of knee OA patients.

Our other studies consistently showed that combined treatment of acupuncture and TENS was more effective than separate treatments [24,25]. TENS was effective for immediate relief of pain, while acupuncture was effective in long-term pain relief and improvement of patients' knee function. In the present study, the A&T group reported lower mean pain intensity VAS scores and lower mean WOMAC scores than other groups.

## Conclusion

The present study clearly demonstrated that combined acupuncture and TENS treatment is effective for pain relief in terms of VAS and knee function improvement in terms of WOMAC in patients suffering from knee OA. Large scale clinical trials are warranted.

## Abbreviations

ACP: acupuncture group; ANOVA: analysis of variance; OA: osteoarthritis; QOL: quality of life; ROM: range of motion; TENS: transcutaneous electrical nerve stimulation; VAS: visual analogue scale; WOMAC: Western Ontario and McMaster Universities Osteoarthritis Index

## Competing interests

The author(s) declare that they have no competing interests.

## Authors' contributions

KI was responsible for study design, acupuncture treatment, manuscript preparation and submission. SH was responsible for patient recruitment. HO was responsible for all statistical design and data analysis. YK was responsible for manuscript review. HK was responsible for study design, critical manuscript review and patient recruitment. All authors read and approved the final manuscript.
